# A study evaluating differences in 3D upper limb kinematics and surface electromyography measures in adults with and without facioscapulohumeral dystrophy

**DOI:** 10.1016/j.xrrt.2026.100670

**Published:** 2026-01-29

**Authors:** Fraser Philp, Martin Seyres, Nicholas Emery, Richa Kulshrestha, Edward K. Chadwick

**Affiliations:** a School of Allied Health Professions and Nursing, University of Liverpool, Liverpool, UK; b Public Health and Sport Sciences, University of Exeter, Exeter, UK; c School of Science and Engineering, University of Dundee, Dundee, UK; d Robert Jones and Agnes Hunt Orthopaedic Hospital Foundation Trust, Oswestry, UK; e School of Engineering, University of Aberdeen, Aberdeen, UK

**Keywords:** Facioscapulohumeral dystrophy, Shoulder, Upper limb, Biomechanics, Electromyography, Motion analysis

## Abstract

**Background:**

Facioscapulohumeral dystrophy (FSHD) is a rare disease that causes progressive muscle wasting and loss of function, with the upper limb being the most affected. Factors leading to loss of arm function are poorly understood. A better understanding of movement profiles determined using 3D movement analysis could help inform treatment selection and development; however, limited evidence is available for combined 3D movement and surface electromyography (sEMG) studies that include the scapula. Our null hypothesis is that there are no differences between the movement and muscle activity of people with FSHD and age- and sex-matched controls (two-tailed).

**Methods:**

Adults were recruited into three groups: (1) FSHD with scapulothoracic arthrodesis (scap fix); (2) FSHD and no surgery (no surgery); or (3) age- and sex- matched control group (CG). Participants attended a single session and carried out seven motion tasks in which their movements and muscle activity was measured using 3D movement analysis and sEMG. Descriptive statistics and normalized movement and muscle activity plots were used to compare joint angles, sEMG patterns, and scapulohumeral rhythm between groups.

**Results:**

Data were collected for 14 participants (10M:4F), seven with FSHD and seven age- and sex- matched controls, with a mean (standard deviation) age of 41.6 (15.7). The FSHD (no surgery) group achieved lower mean (standard deviation) thoracohumeral elevation, most notably in flexion, 74.6° (29.2), and abduction 80.8° (31.2), compared to the CG, who achieved 126.9° (12.7) and 130.1° (10.8), respectively Despite these differences, range of movement for glenohumeral elevation was similar between groups. Considerable variability across the acromioclavicular and sternoclavicular joints was noted in all FSHD groups, with no clear between group differences. Scapulohumeral rhythm was reduced in the FSHD (no surgery) group. FSHD groups demonstrated prolonged and higher normalised activity levels of the trapezius, anterior deltoid, and infraspinatus muscles. This was most evident during the middle of the motion being carried out.

**Discussion:**

Evaluations that focus on arm position alone are insufficient for explaining why people with FSHD lose arm function. People with FSHD had lower thoracohumeral elevation angles compared to the CG, and the limited elevation was a result of altered scapula rather than glenohumeral joint kinematics. Timing and normalized sEMG levels for the FSHD group was variable, with no clear between-group differences. The scapular and muscle activity patterns observed in the FSHD group were heterogenous, which made identification of between groups difficult in our limited sample size.

**Conclusion:**

People with FSHD demonstrated limited arm movements primarily from altered scapular kinematics. The scapular and muscle activity patterns observed in the FSHD group were heterogenous which made identification of between groups difficult in our limited sample size.

Facioscapulohumeral dystrophy (FSHD) is a rare muscle disease that causes progressive muscle damage, wasting and loss of function.^26^^,^^29^^,^^35^ The presentation and progression of the disease is highly variable and asymmetric between individuals. Despite this, changes to the morphology of muscles which control movements of the scapular and shoulder girdle is one of the most prevalent and apparent consequences.^5^ This can result in altered scapular kinematics and biomechanical changes to the glenohumeral joint (GHJ), which present alongside limited shoulder range of movement and pain.^4^^,^^6^^,^^13^^,^^31^ People with FSHD can experience difficulties with functional tasks such as reaching, lifting, carrying objects and management of personal care.^5^^,^^6^ Maintaining arm elevation is important as reduced arm function is linked to a loss of independence and participation, which negatively impacts quality of life.^4^^,^^6^

Establishing large scale effectiveness trials in rare disease subtypes such as FSHD is challenging.^24^ Currently, there is limited evidence to inform management of the upper limb and engagement with exercise-based rehabilitation is low.^13^^,^^24^ Some people with FSHD may be offered surgical fixation of their scapula to reduce pain or increase arm elevation. However, these benefits diminish over time, long-term outcomes are variable, and some people experience complications.^2^^,^^11^^,^^15^^,^^22^ In the absence of data derived from robust clinical trials, movement analysis may provide an alternative method for evaluating effectiveness and the disease mechanism.^11^^,^^15^

Movement analysis is already used to inform clinical decision making in other rare diseases;^39^ however, a lack of standard reference tasks and protocols for assessing upper limb movements is known to be a barrier to wider use in clinical practice.^30^ Previous research has used movement analysis to evaluate the movement^7^ and control strategies^12^ of people with FSHD. This has involved 3-dimensional (3D) kinematic analysis or surface electromyography (sEMG).^3^^,^^7^^,^^11^^,^^12^^,^^34^ Studies have identified that people with FSHD have slower movement speeds (between 25 and 40%)^3^^,^^7^ and increased elbow flexion (possibly to reduce the overall moment at the shoulder),^3^^,^^7^ in addition to increased levels of muscle activation, compared to controls.^3^^,^^7^^,^^11^^,^^12^^,^^34^ Movement analysis studies investigating the biomechanical effects of scapulothoracic (TS) fixation are limited.^2^ While these studies have provided valuable information to the field, there are still some gaps in our current understanding. Existing studies have either only included people with higher levels of arm function (Brooke scale ≥4),^7^^,^^12^ assessed a limited number of movements,^3^^,^^12^ not combined assessment of 3D joint angles with sEMG,^34^ used simple kinematic measures^3^^,^^11^ or models which did not include the scapula,^7^^,^^12^ or modeled simplified representations of the shoulder complex eg TS and thoracolumbar segments only.^7^^,^^12^^,^^34^ While other measures of arm function such as reachable workspace provide useful information regarding an individual's global level of arm function, they do not always provide information for individual joints or muscles of the shoulder girdle, which may be required for understanding disease mechanisms or tailoring interventions.^14^^,^^15^^,^^25^^,^^28^ Accepted conventions that allow for the identification of deviations within the condition and clear links between the activity, impairment, and intervention are required for informing decision making.

Further evidence for movement and muscle activity pattern differences in people with FSHD, based on combined sEMG and more detailed kinematic representations of the shoulder girdle, which include scapular movement is needed. The aim of this study was therefore to quantify the movement and muscle activity patterns of people with and without FSHD using 3D motion capture, sEMG and a physiologically representative model of the shoulder girdle which includes scapula kinematics.

## Materials and methods

Ethical approval for this study was gained from the West Midlands - Black Country Research Ethics Committee 21/WM/0275. This trial is registered on ClinicalTrials.gov Identifier: NCT05239520 https://classic.clinicaltrials.gov/ct2/show/NCT05239520.

### Study design

This is an exploratory cross-sectional, case control study. An overview of the protocol and results from a previous study based on this dataset has been reported previously.^32^ As this was an exploratory study, and to explore the differences in movements across groups, convenience sampling of people with FSHD and varying levels of arm function, surgical (scap fix) and nonsurgical interventions (no surgery) was used. The control group (CG) was age- and sex-matched. Participants for all groups were recruited from a single tertiary center and through advertising specialist regional centers.

A formal sample size calculation was not conducted as this is an exploratory cross-sectional study. The sample size was consistent with previous similar studies and is appropriate for the selected methods of analysis.^7^^,^^19^

Informed consent was gained from all participants who attended the single measurement session. Demographic, clinical, 2-dimensional ultrasound and 3D joint and sEMG measures of the upper limb was conducted. The 2-dimensional ultrasound measures were taken prior to the 3D movement analysis session.^32^

### Inclusion criteria

People older than 18 years of age were included for both groups. A confirmed diagnosis of FSHD was required for the FSHD group and stratified sampling based on arm function and previous surgery was used.

### Exclusion criteria

FSHD participants with any recent trauma to the shoulder within the last 3 months that had not resolved, surgery to the thorax or upper limb in the last 6 months, a previous history of fracture to the shoulder joint or any coexisting neurological pathologies or additional musculoskeletal injuries to the upper limb being assessed were excluded. CG participants with any previous presentation to a health care professional with a diagnosis of shoulder instability, a previous shoulder injury within the last three months that had not resolved, any coexisting neurological pathologies or deficits, any previous surgical interventions on the arm or were undergoing or awaiting medical management, diagnostic investigations on the arm were excluded.

### Demographics and clinical assessments

Patient demographics, Beighton scores of hypermobility and grip strength testing were recorded in addition to a clinical assessment of the shoulder.^32^

### Three-dimensional motion analysis protocol

3D motion analysis was carried out according to a published protocol, used in young people with shoulder instability.^37^ A full detailed overview of the marker cluster, sEMG placement, static calibration processes, gap filling and filtering of kinematic and surface electromyography waveforms have been reported there.^37^ Clusters comprising passive retroreflective markers were placed on the thorax, acromion, humerus, forearm, and hand segments.^20^^,^^21^^,^^37^^,^^40^ Available at https://doi.org/10.17638/datacat.liverpool.ac.uk/2386. The clusters are used to define local coordinate frames for each segment, in which the coordinates of bony landmarks (virtual markers) are defined.^37^ This enables continuous tracking of relevant bony landmarks (e.g. Humeral epicondyles) throughout dynamic motions. sEMG electrodes were placed on the middle trapezius, infraspinatus, triceps, latissimus dorsi, deltoid (posterior and anterior), pectoralis major, biceps, wrist flexor and extensor muscles consistent with SENIAM guidelines^16^ and Criswell et al.^9^

Participants completed all movements while seated and carried out 4 unweighted upper-limb tasks (flexion, abduction, abduction to 45° with axial rotation, and hand to back of head) and 3 weighted tasks (self-selected) of 0.5 kg, 1.0 kg or 1.5 kg (flexion, abduction, abduction to 45° with axial rotation) in that order. A total of 12 repetitions (2 sets x 6 repetitions) were carried out for unweighted tasks, and a total of 6 repetitions (2 sets x 3 repetitions) were carried out for weighted tasks.

An assessor initially demonstrated movements to participants, who were then asked to carry them out to a count of 3 seconds up, 3 seconds down as able. During the tasks, the assessor was seated in front of participants to enable mirroring of the movements.

A 12 camera (V5-Vantage) Vicon motion capture system, with a sampling frequency of 100 Hz, and 2 synchronous sagittal and coronal video recordings was used for recording movements. Gap filling for marker data was carried out using rigid body, pattern and spline filling pipelines as required in Vicon Nexus 2.12.1.^41^ A Delsys Trigno electromyography system, with sampling frequency of 2000 Hz was used for measuring muscle activity. Quality control methods for evaluating electrode placement, signal quality and carrying out of submaximal movements were conducted.^37^

### Data processing and analysis

The Wu shoulder model^44^ was used for calculating joint kinematics in OpenSim 4.4 (National Center for Simulation in Rehabilitation Research Stanford University, Palo Alto, CA, USA) using the Inverse Kinematics tool. With this tool, following model scaling, a global optimization is carried out to minimize the errors between measured marker positions and the corresponding markers defined in the model. This results in an estimate of joint kinematics that best fits the measured data.^10^^,^^36^^,^^43^ The model is comprised of 5 segments (thorax, clavicle, scapula, humerus, and combined forearm/hand), with 10 degrees of freedom (DOF) ie GHJ and acromioclavicular joints (ACJs) modeled as 3-DOF constrained ball and socket joints, and the sternoclavicular (SC) and elbow joints modeled as a 2-DOF universal joints.

International Society of Biomechanics (ISB) recommendations for joint co-ordinate system definitions were followed.^43^ While conventions used in our study are consistent with ISB recommendations and terminology, differences in terms exist across studies and models. To further help with interpretation, definitions of the angles associated with the ACJ have been provided. For the ACJ, retraction and protraction of the scapula occurs about an axis aligned with the vertical axis of the clavicle; anterior or posterior tilt about the mediolateral axis of the scapula; external or internal rotation about an axis orthogonal to the above 2 (ie approximately anteroposterior). This is referred to as lateral or medial rotation in the ISB standards.^43^

Scaling ratios from marker pairs associated with individual bony segments identified during static calibration and movement waveforms produced by inverse kinematics were reviewed for all participants and trials, consistent with best practice.^17^^,^^36^^,^^37^

Data from all available repetitions were time normalized and averaged for the analysis. A Savitzky-Golay filter, with a window size of 99 and a polynomial order of 2 was used for kinematic smoothing.^44^ Filter and parameters selections were based on demonstrably superior performance on our data set for removal of noise while preserving the underlying signal. This is consistent with studies demonstrating improved performance during high-frequency acceleration-time signals when compared to alternative methods.^33^

Geometrical scaling was used for determining the GHJ center. While not inherent in the selected model, thoracohumeral (TH) and TS angles were generated for evaluation of scapulohumeral rhythm and to reflect a clinician's observation in practice. Thoracohumeral elevation angle was calculated to identify arm position. Joint-specific angles for the GHJ, SC joints, and ACJs were also calculated. Scapula vs. TH angles were calculated for the activities of unweighted flexion and abduction to evaluate scapulohumeral rhythm.

A band-pass, second-order Butterworth filter of 10-400 Hz and zero lag correction was applied to sEMG signals.^42^ Maximum voluntary contraction testing was not carried out as this is known to have limited reliability and high variability in FSHD and other pathological populations.^7^^,^^38^ sEMG normalization was carried out against the maximum encountered activation across any of the movement activities. This included isolated movements against resistance for quality control, grip, weighted and unweighted tasks was used for normalization of sEMG signals.^27^

C3D files used for 3D movement and sEMG analysis are available at: https://doi.org/10.17638/datacat.liverpool.ac.uk%2F2600.

Group demographics and joint angles are reported using descriptive statistics. A subset of joint angles and sEMG activity for CG and FSHD (no surgery) participants have been plotted graphically for the respective movement tasks. No formal statistical comparisons were made due to the sample size and heterogeneity of the FSHD group to mitigate inference errors.

## Results

Data were collected for a total of 14 participants. This included 7 people with FSHD and 7 age- and sex-matched controls. Demographic characteristics for all participants are presented in Table I. The FSHD (scap fix) group consisted of 2 participants with previous TS arthrodesis.

**Table I:** Demographic characteristics of study participants.

Demographic and clinical characteristics of study participants	Age-matched controls (CG) (n = 7)	People with FSHD combined (n = 7)	People with FSHD and no surgery (no surgery) (n = 5)	People with FSHD and previous scapulothoracic arthrodesis (scap fix) (n = 2)
Age (yr)	41.3 (15.5)	41.9 (17.1)	48.0 (16.3)	26.5 range (23-30)
Sex (M:F)	5:2	5:2	4:1	1:1
Height (median (IQR))	176.4 (5.7)	176.0 (8.8)	178.2 (6.5)	170.6 range (160.2-181)
Weight (mean (SD))	77.1 (11.2)	90.6 (24.8)	96.8 (26.7)	75.1 range (66.2-84.0)
Beighton score (median (IQR))	1 (0-2)	0 (0-1.5)∗	0 (0-1)∗	1 range (0-2)
Dominant hand (L:R)	(1:6)	(2:4)	(2:3)	(0:2)
Weight selection for loaded tasks (0.5 kg:1.0 kg:1.5 kg)	(0:0:7)	(1:4:2)	(1:2:2)	(0:2:0)

*FSHD*, facioscapulohumeral dystrophy; *CG*, control group; *SD*, standard deviation; *IQR*, interquartile range.

∗ Two participants unable to complete Beighton due to standing balance issues.

### Joint kinematics

An overview of all joint kinematics for all and participants is provided in Table II. Mean and standard deviation (SD) values are provided for CG and FSHD (no surgery) participants, whereas the range of mean values for individual people with FSHD (scap fix) group are shown.

**Table II:** ROM values for all participants, movements and joints (degrees).

Motion	Flexion			Flexion with weight			Abduction			Abduction with weight			Abduction at 45° with axial rotation			Abduction to 45° with axial rotation and weight			Hand to back of head		
Group	CG-mean (SD)	FSHD (no surgery) – mean (SD)	FSHD (scap fix) range	CG	FSHD (no surgery)	FSHD (scap fix)	CG	FSHD (no surgery)	FSHD (scap fix)	CG	FSHD (no surgery)	FSHD (scap fix)	CG	FSHD (no surgery)	FSHD (scap fix)	CG	FSHD (no surgery)	FSHD (scap fix)	CG	FSHD (no surgery)	FSHD (scap fix)
TH elevation plane	78.4 (14.1)	72.6 (4.5)	56.9-86.8	77.1 (13.3)	72.3 (2.4)	52.4-70.4	76.1 (24.9)	49.2 (23.7)	12.6-65.6	74.8 (25.5)	45.5 (19.5)	23.5-43.5	15.4 (8.4)	19.5 (5.8)	15.8-20.8	16.8 (11.9)	25.7 (10.2)	10.0-19.4	57.4 (29.1)	48.8 (4.8)	30.6-94.6
TH elevation angle	126.9 (12.7)	74.6 (29.2)	59.9-99.7	128.1 (11.4)	71.4 (36.6)	49.3-97.8	130.1 (10.8)	80.8 (31.2)	50.8-106.6	131.2 (10.9)	80.4 (32.1)	35.9-93.9	14.7 (6.7)	12.8 (3.4)	8.1-12.3	16.3 (5.3)	15.1 (3.8)	13.5-18.3	106.6 (7.6)	69.8 (24.5)	56.6-96.1
TH rotation	73.2 (12.3)	78.1 (8.4)	41.2-87.2	75.2 (7.6)	76.0 (9.2)	65.9-72.1	78.3 (32.1)	61.9 (22.2)	41.6-67.9	76.0 (31.7)	58.0 (10.8)	40.2-43.7	99.2 (23.0)	73.2 (8.0)	41.1-79.4	94.9 (20.8)	79.4 (15.4)	44.1-69.2	84.2 (19.3)	67.2 (8.2)	55.4-85.5
TS protraction	19.7 (7.0)	14.1 (5.0)	4.2-13.1	23.0 (7.3)	12.8 (3.0)	4.2-18.9	20.7 (10.0)	19.0 (9.9)	4.8-11.9	21.7 (12.4)	19.6 (8.0)	3.0-7.8	10.5 (5.9)	9.4 (4.5)	5.4-6.9	11.2 (5.3)	10.1 (4.6)	7.3-10.1	12.9 (7.8)	11.7 (6.4)	19.0-20.7
TS rotation	41.3 (7.4)	25.5 (11.8)	10.0-21.3	44.8 (9.5)	28.8 (12.2)	10.9-24.2	43.8 (10.5)	23.6 (12.6)	3.0-18.5	45.8 (11.9)	25.6 (15.3)	3.9-11.7	11.4 (4.3)	9.1 (4.1)	2.6-3.1	12.9 (3.5)	11.0 (3.4)	3.3-4.9	33.1 (9.3)	19.3 (13.8)	6.9-20.5
TS tilt	23.0 (10.3)	25.7 (7.2)	9.5-17.1	24.8 (9.1)	26.0 (4.3)	9.5-19.4	18.6 (5.0)	20.3 (6.5)	3.6-20.8	15.1 (4.3)	21.4 (7.8)	3.2-19.5	10.6 (5.2)	10.6 (4.3)	4.7-12.0	12.6 (6.5)	11.6 (4.5)	10.5-12.6	19.5 (7.4)	18.9 (6.6)	15.1-15.1
GHJ elevation plane	61.4 (32.6)	46.2 (13.3)	20.8-90.6	53.4 (16.4)	54.4 (26.5)	32.3-65.6	50.0 (13.4)	41.9 (16.6)	27.0-27.8	44.7 (9.9)	41.8 (20.1)	11.9-15.1	9.2 (8.2)	15.4 (6.3)	12.3-12.5	9.5 (7.4)	13.9 (8.0)	2.7-17.3	31.1 (15.4)	28.7 (9.9)	35.8-84.8
GHJ elevation angle	91.4 (11.7)	76.2 (6.6)	64.7-99.1	91.5 (13.0)	77.8 (11.3)	55.5-88.0	94.5 (12.1)	72.7 (12.6)	48.9-88.7	92.5 (13.7)	70.1 (11.7)	42.2-80.5	17.1 (5.4)	15.1 (5.5)	11.0-13.6	15.5 (4.9)	16.2 (6.8)	10.7-18.4	77.8 (11.5)	71.7 (6.7)	61.4-91.8
GHJ rotation internal/external	83.8 (29.5)	80.6 (11.9)	21.1-93.7	80.7 (20.1)	77.7 (17.7)	41.9-82.6	59.5 (15.1)	46.2 (13.8)	34.7-52.4	53.3 (13.7)	44.2 (15.0)	50.6-51.5	100.3 (11.9)	77.6 (14.1)	60.6-86.9	89.0 (15.5)	83.6 (19.3)	51.9-79.7	71.8 (21.2)	63.8 (9.2)	45.5-97.5
ACJ tilt	44.0 (19.0)	38.0 (25.6)	11.6-22.2	46.2 (19.3)	36.7 (22.5)	10.8-23.9	45.5 (13.0)	33.2 (24.0)	3.9-24.5	43.4 (12.4)	34.7 (25.4)	3.1-19.3	10.9 (4.4)	8.4 (5.2)	4.1-12.7	14.0 (4.7)	10.5 (5.0)	9.6-14.2	38.3 (15.7)	28.8 (23.9)	18.0-18.7
ACJ protraction/retraction	12.8 (8.2)	13.8 (5.5)	6.0-12.8	14.1 (10.1)	14.2 (6.4)	5.9-11.7	17.4 (10.5)	11.7 (3.4)	3.3-11.5	17.0 (10.2)	11.4 (2.3)	3.0-11.1	8.7 (3.2)	8.9 (5.8)	6.6-7.9	9.5 (2.7)	8.2 (5.3)	10.0-11.3	9.4 (5.7)	14.5 (4.8)	6.4-7.7
ACJ rotation internal/external	25.5 (10.0)	13.4 (2.2)	10.1-20.5	26.9 (11.1)	14.1 (3.1)	9.5-20.9	26.1 (14.8)	12.7 (4.2)	2.8-19.5	26.1 (15.9)	13.1 (3.2)	2.4-15.5	7.4 (2.5)	5.2 (1.7)	2.8-4.9	7.2 (3.6)	6.0 (1.9)	4.3-5.9	17.9 (8.0)	10.6 (2.8)	2.9-18.3
SCJ protraction/retraction	19.1 (11.1)	14.3 (5.8)	5.3-13.5	21.1 (11.0)	15.7 (6.6)	5.0-14.8	20.5 (11.4)	17.0 (6.9)	6.4-15.6	21.7 (11.3)	17.7 (6.8)	3.6-12.4	6.5 (3.7)	6.2 (4.2)	2.9-3.4	7.7 (3.7)	7.2 (3.6)	2.9-3.6	17.8 (8.1)	17.6 (5.1)	11.6-16.5
SCJ elevation/depression	14.7 (5.5)	12.0 (6.8)	4.1-5.5	15.9 (6.6)	13.9 (7.5)	3.9-5.3	16.3 (7.7)	12.3 (9.3)	2.6-5.7	16.8 (7.2)	13.3 (10.9)	2.3-5.4	4.2 (2.3)	4.3 (2.5)	1.8-3.4	5.4 (3.1)	5.6 (3.0)	1.9-4.9	12.8 (6.2)	11.2 (7.6)	4.3-8.2

*TH*, thoracohumeral joint; *TS*, scapulothoracic; *GHJ*, glenohumeral joint; *ACJ*, acromioclavicular joint; *SCJ*, sternoclavicular joint; *FSHD*, facioscapulohumeral dystrophy; *SD*, standard deviation.

FSHD (scap fix): people with FSHD and previous scapular arthrodesis surgery; FSHD (no surgery): people with FSHD and no scapular arthrodesis; CG represents age- and sex-matched control group.

**Figure 1:**
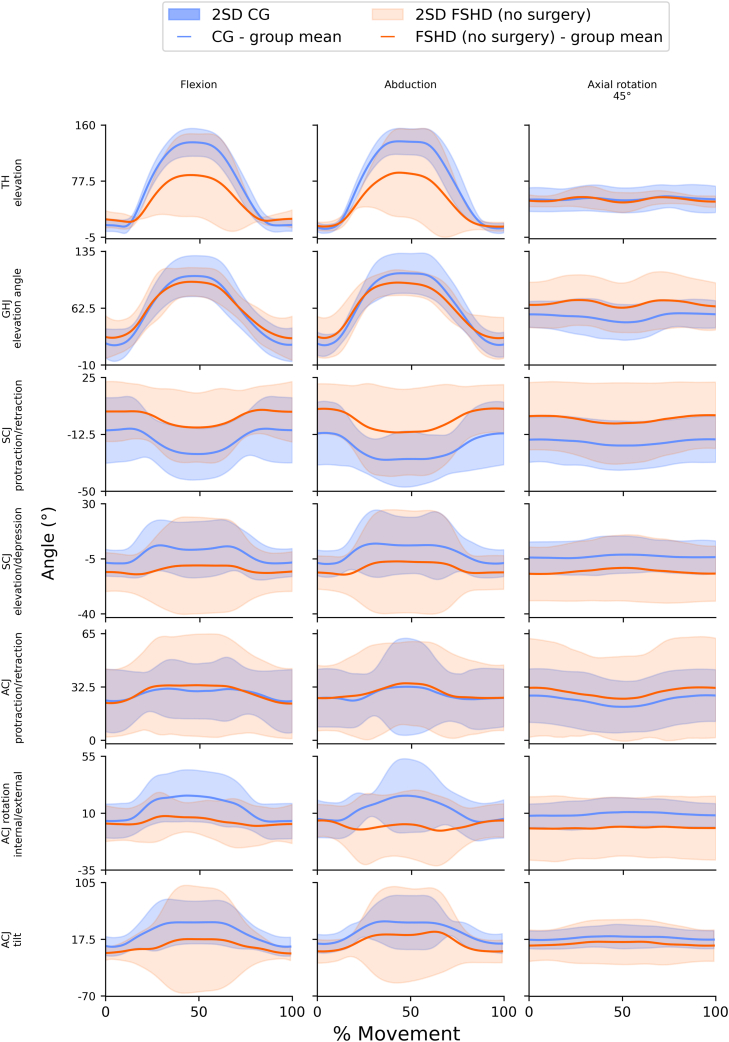
Subset of plots demonstrating differences in arm elevation and the contribution of individual joints for the FSHD (no surgery) and CG groups. Joint angles for relevant joints and movements. Lines show mean group angles. Shaded areas indicate the 2SD. 2SD, 2 standard deviations. Column headings key: Axial rotation 45°: abduction at 45° with axial rotation; FSHD (no surgery): people with FSHD and no scapular arthrodesis; *CG*: age- and sex-matched control group. *TH*, thoracohumeral joint; *GHJ*, glenohumeral joint; *SCJ*, sternoclavicular joint; *ACJ*, acromioclavicular joint; *FSHD*, facioscapulohumeral dystrophy; *CG*, control group.

A subset of plots demonstrating differences in arm elevation and the contribution of individual joints for the CG and FSHD (no surgery) participants is presented in Figure 1. Plots show mean range of motion and 2 SDs. An overview of plots for all joints, movements, and participants is available in Supplementary Appendix S1.

Overall, the FSHD (no surgery) group had lower TH elevation angles than the CG across almost all tasks. This was most evident in the unweighted and weighted movements of flexion and abduction with mean differences of approximately 50° between groups. Range of movement for glenohumeral elevation was similar between the FSHD (no surgery) and CG, with the FSHD (no surgery) having a slightly lower mean ROM values for unweighted weighted flexion 76.2° (6.6) vs. 91.4° (11.7) and unweighted abduction 72.7° (12.6) vs. 94.5° (12.1). Apart from the movements of unweighted and weighted flexion and abduction, the glenohumeral elevation plane was similar between the FSHD (no surgery) and CG with values of 46.2° (13.3) and 41.9° (16.6) vs. 61.4° (32.6) and 50.0° (13.4) respectively. Variations in the glenohumeral elevation plane for these motions may be a result of FSHD (no surgery) participants adjusting the position of the arm to achieve the task. As demonstrated in the plots the FSHD (no surgery) group had more ACJ tilt and less ACJ rotation compared to the CG. Both SC protraction and elevation were higher for the FSHD (no surgery) group compared to the CG. The 2SD band for the ACJ and SC joints in people with FSHD (no surgery) were very wide, indicating considerable variability in the movements of individuals. This suggests the limitation in people with FSHD's ability to elevate their arm is more likely a consequence of altered scapula kinematics rather than limitations at the GHJ. FSHD (scap fix) participants had minimal movement across the ACJ and SCJ consistent with the intended purpose of the surgery.

### Scapulohumeral rhythm

**Figure 2:**
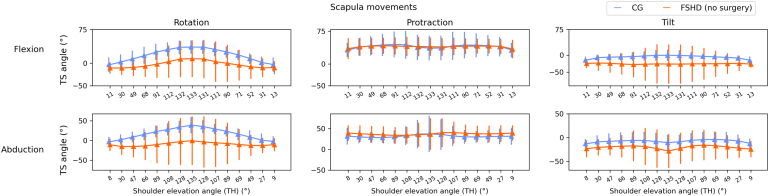
Scapulohumeral rhythm plots (scapula versus thoracohumeral elevation angle plots) for the movements of unweighted flexion and abduction. *TS*, scapulothoracic; *TH*, thoracohumeral; *FSHD*, facioscapulohumeral dystrophy; *CG*, control group.

The FSHD (no surgery) group had lower scapula rotation through arm elevation compared to the CG, although variability within the FSHD (no surgery) group was high as shown in Figure 2. This was also true for scapula tilt measurements. For flexion, the CG and FSHD (no surgery) groups demonstrated similar scapulohumeral rhythm for scapula protraction relative to arm elevation. For abduction, people with FSHD (no surgery) had higher scapula protraction angles than the CG when the arm was elevated below 90°, above which values/patterns become more similar. These results demonstrate that the FSHD (no surgery) group have a reduced but more variable scapulohumeral rhythm (rotation and tilt) compared to CG participants. These are reflective of the patterns observed in the individual joint angle plots.

### Muscle activity profiles

**Figure 3:**
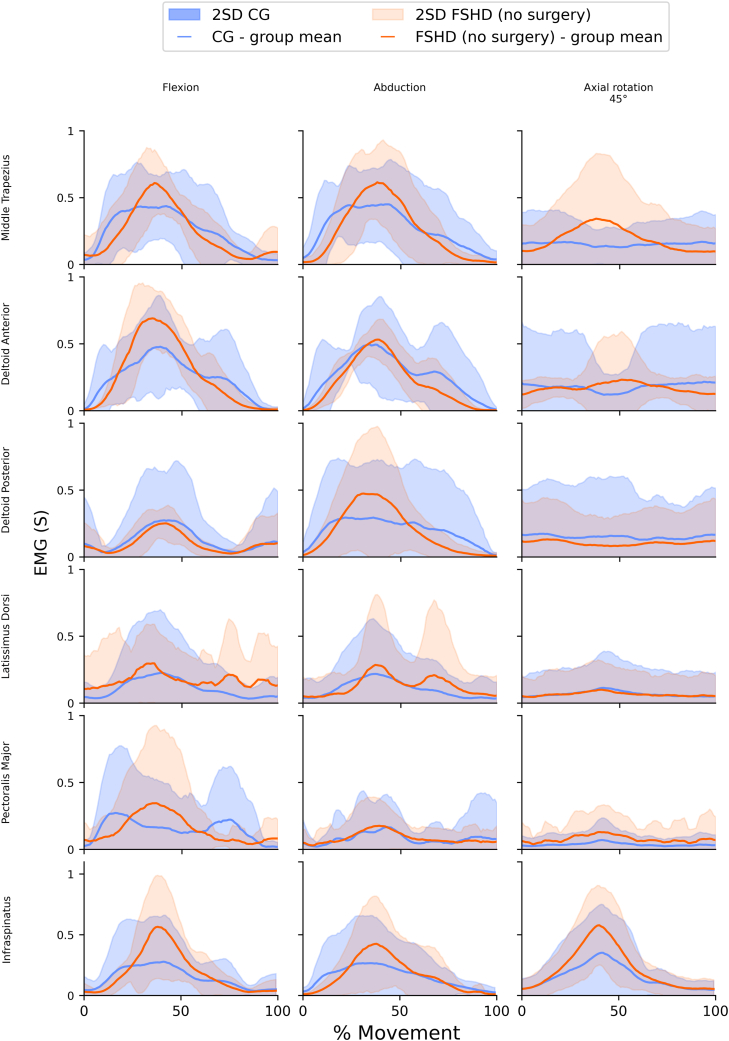
A subset of plots showing muscle activity for the shoulder girdle muscles in the FSHD and CG groups. Surface EMG for all movements. Lines show mean normalized signals. Shaded areas indicate the 2SD. 2SD, 2 standard deviations. Column headings key: Axial rotation 45°: abduction at 45° with axial rotation; FSHD (No surgery) people with FSHD and no scapular arthrodesis; CG: age- and sex-matched control group. *EMG*, electromyography; *FSHD*, facioscapulohumeral dystrophy; *CG*, control group.

A subset of plots demonstrating differences in muscle activity differences in CG and FSHD (no surgery) groups is presented in Figure 3. An overview of plots for all muscles, movements and participants is available in Supplementary Appendix S2.

Several differences in the pattern, timing and level of normalized activity of electromyography values between people with FSHD (no surgery) and the CG are evident. While these are dependent on the movement being carried out, some common features were identified. Broadly it appears that for most tasks, the FSHD (no surgery) groups had higher normalized muscle activity levels, usually in the middle of the movement cycle compared to the CG. This was most notable in the trapezius, anterior deltoid and infraspinatus muscle groups. Increased activity in the FSHD (no surgery) group, for posterior deltoid was only seen during the weighted and unweighted abduction movements. Similarly increased activity for the FSHD (no surgery) group, for pectoralis major was mainly seen in weighted and unweighted flexion movements. Latissimus dorsi activity for the FSHD (no surgery) group displayed a more distinct ‘double peak’ waveform, most evident in the movements of flexion, abduction and axial rotation with weight activities compared to the CG.

## Discussion

The aim of this study was to quantify the movement and muscle activity patterns of people with and without FSHD using 3D motion capture, sEMG and a physiologically representative model of the shoulder girdle that included scapula kinematics. Our study demonstrated that people with FSHD had lower TH elevation angles compared to the CG and that the limited elevation was a result of altered scapula rather than GHJ kinematics. This was most evident in the FSHD (no surgery) group who presented with decreased SC protraction and elevation alongside decreased ACJ upward rotation and tilt. A direct comparison of 3D joint angles and sEMG data between our study and other research is not possible due to differences in the sample demographics, movements assessed, biomechanical models used (which informs the number of segments), muscles measured, and methods of analysis used for joint kinematics and sEMG eg normalization. However, some common features between studies have been identified.

### Joint kinematics

Participants with FSHD demonstrated lower TH elevation angles compared to the CG (approximate mean difference of 50°), consistent with previous research.^7^^,^^12^^,^^34^ Essers et al 2019^12^ evaluated TH angles and sEMG in people with FSHD and higher levels of arm function (Brooke scale 3 to 4). Overall people with FSHD achieved lower levels of mean (SD) shoulder elevation in flexion (83° (20) vs. 141° (18)) and shoulder abduction movements (70° (18) vs. 149° (19)) compared to the control group. Bergsma et al^7^ also evaluated TH angles and sEMG across a range of functional and planar movements including shoulder flexion and abduction (Brooke scale 2 to 3). People with FSHD achieved lower median (interquartile range) shoulder elevation angles compared to the control group for flexion 65.8° (60.5–81.3) vs. 144.9° (141.0–145.9) and abduction 81.6° (67.6–96.3) vs. 147.6° (144.8–149.7). Demirci et al^34^ identified that people with FSHD had lower TH elevation angles, with none achieving more than 120°. Lower levels of arm elevation for people with FSHD is anticipated based on the natural history of the condition and sampling frame used across studies. Similar to our study, only Demirci et al^34^ measured scapular kinematics and identified that less elevation was associated with altered scapular kinematics.

Scapula movement and the associated scapulohumeral rhythm had a bigger contribution to lost elevation compared to movement at the GHJ in people with FSHD. While there was considerable variability in the scapula position across movements, for tasks involving large amounts of elevation, the FSHD (no surgery) group demonstrated decreased SC protraction and elevation alongside decreased ACJ upward rotation and tilt. Evaluation of scapulohumeral rhythm during flexion and abduction, also identified reduced rotation of the scapula in all FSHD groups. This is consistent with Demirci et al^34^ who identified that FSHD participants had more scapula downwards rotation and increased posterior scapula tilt, although this was less consistent throughout the overall elevation movement as identified in our study. While differences were seen within the scapula rotation and tilt planes, differences in overall patterns of movement between our study and Demirci et al^34^ may stem from differences in the study demographics due to the heterogeneous nature of FSHD, and differences in the epochs of thoracolumbar elevation used to assess scapulohumeral rhythm. Our findings confirm those of Demirci et al^34^ and provide data that supports the use of interventions that effectively support stability and encourage upward rotation of the scapula which may enhance or maintain arm elevation. This is consistent with current clinical reasoning used in surgical interventions such as TS arthrodesis, or use of braces or supports.^11^

As anticipated, participants with FSHD, and previous scapular arthrodesis had limited ACJ and SJ movement, consistent with the intended purpose of the surgery. Use of FSHD (scap fix) participants in this study allowed for an evaluation of the biomechanical models face validity. This is important, as inverse kinematics, the process that uses a global optimization to minimize the errors between measured and model defined marker positions to estimate joint kinematics,^10^^,^^36^^,^^43^ may not always provide appropriate solutions. One FSHD (scap fix) participant had similar movement in the ACJ tilt and SCJ protraction/retraction planes to that of the FSHD (no surgery) group. This likely stems from soft tissue artefact given the nature of the surgery.^8^^,^^40^ This highlights the need to interpret the derived data with sufficient understanding of the capture and analysis processes. Biomechanical models, with varying levels of personalization, have been used for surgical planning in other patient groups.^1^^,^^23^ For these methods to be used more widely in clinical practice, knowledge of possible sources and limits of error are needed.^39^ This study provides preliminary data that may inform the steps required for further translation.

### Muscle activity levels

People with FSHD demonstrated higher normalized activity levels of the shoulder girdle muscles compared to the CG. This was usually in the middle of the movement cycle and more consistently seen in the trapezius, anterior deltoid and infraspinatus muscle groups. Increased activity in the trapezius, biceps, deltoid, pectoralis and serratus anterior muscle groups have consistently been identified in previous research.^3^^,^^7^^,^^12^ The higher sEMG activity observed is likely due to structural changes caused by the condition which limits the muscles force generating capacity. Some muscles may be unable to stabilize or mobilize the required segments sufficiently, and to accommodate this, the neuromuscular system may increase the overall level and duration of the activity, alongside recruitment of alternative muscles. This is consistent with the existing hypothesis that people with FSHD have less intact active muscle tissue, and will therefore recruit a higher percentage of the remaining muscle fibers to perform tasks.^3^ This may also have a higher metabolic cost and could contribute to feelings of fatigue, which is often experienced by people with FSHD.^18^

Understanding why people adopt different movement strategies and the implications may be helpful within a rehabilitation context. Differences in sEMG may stem from differences in the arm position for people with FSHD who modify their limb position to achieve a mechanical advantage.^3^ For example, in our study the FSHD group had increased posterior deltoid activity during the unweighted abduction movement task, and different (lower) plane of elevation values. Rehabilitation philosophies that center around imposition of an ‘ideal’ movement strategy for task completion may therefore need to be considerate of reasons for the observed differences.

Unlike some studies, we did not use maximum voluntary contractions for normalization but still identified similar differences across the same muscle groups. Bergsma et al (2014) reported normalized activity values of 115% in the trapezius muscle of people with FSHD and reflects a possible consideration when using this method.^7^ No method of normalization is without limitations, particularly in pathological populations, but selection of an appropriate normalization method may allow for longitudinal changes to be observed which may provide further insight into adaptations of the musculoskeletal system over the natural history of the condition.

### Movement variability and feature identification

Overall, the FSHD group demonstrated more variable 3D joint kinematic and sEMG patterns. This is consistent with the heterogeneous nature of the disease and patterns observed in other studies.^3^^,^^7^^,^^12^ sEMG patterns observed in our study were highly variable both within and between tasks, which makes identification of differences difficult, similar to previous studies.^12^ People with FSHD may be adopting movement strategies that allow them to undertake movements and optimizing them based on the biomechanical changes and subsequent constraints of the neuromuscular system.^7^^,^^11^^,^^12^^,^^34^^,^^41^

Similar to our previous research, assessment of upper limb function based on 3D movement analysis and sEMG produces large amounts of data.^37^ Loss of arm elevation was evident in our dataset and future protocols may choose to focus in more detail on this. While alternative methods such as reachable workspace and overall arm position are available, they do not provide joint specific information which may be helpful for better targeted interventions. Our study demonstrates that additional kinematic data for the relevant shoulder girdle segments in combination with sEMG can be helpful for identifying causes for limited arm movements.

Further evaluation of the relationship between muscle structure changes observed using imaging and sEMG patterns may assist with interpretation of patterns observed and provide further data in support of existing hypothesis.^32^ It is important not to over emphasize the findings of differences observed for sEMG both within and between studies, as these are likely a function of the muscles and movements measured. Interpreting mechanistic relationships between kinematic and sEMG measurements, comparing movement patterns between groups, characterizing causes of heterogeneity or identifying novel biomechanical features of FSHD requires more detailed data analysis and larger sample sizes.

### Limitations

The scapular and muscle activity patterns observed in the FSHD group were heterogenous which made identification of between groups difficult in our limited sample size. As a result of our small sample size, no formal statistical comparisons were carried out to mitigate inference errors. Differences identified in our study are therefore subject to visual observations and narrative descriptions.

This was a single measurement study. Inferences regarding the role of the joint and muscle patterns on the observed differences and possible longitudinal causes of reduced arm function should be interpreted with this understanding. Our measurement protocol also evaluated a limited number of movements and superficial muscles. Furthermore, our inclusion criteria required participants to be able to complete the entire protocol. Similar evaluations of arm function based on this protocol is unlikely feasible in those with less overall arm function. Differences observed are therefore relative to the movement protocol. Deeper muscles such as serratus anterior are known to be affected in people with FSHD and have an impact on shoulder function. Further longitudinal data in a larger sample of people with FSHD and variable arm function are needed.

Inherent to our selected method of normalization, the denominator for normalization of the movement may have arisen from different movement tasks. While this was broadly consistent between participants, selection of a different method may alter the results observed. Irrespective of this, there was some overall increased activity in the muscle groups assessed in our study and other studies. As identified no normalization method is without limitations. sEMG placement was carried out by trained experts and according to established protocols, guidelines and quality control checks which should mitigate the occurrence of crosstalk or artefactual recordings.

While overall people with FSHD have lower TH elevation angles, participants still moved through a wide range of motion. Established modeling conventions were used for analysis, however, shoulder joint angles calculated at these ranges, while mathematically accurate, may not align with expected clinical observations. Interpretation of derived joint angles should be carried out with this understanding.

## Conclusion

People with FSHD demonstrate highly variable movement and muscle activity profiles compared to CG participants. People with FSHD achieved lower thoraco humeral elevation angles consistent with the condition. Reduced overall shoulder elevation stemmed from increased ACJ tilt and reduced ACJ rotation. Both SC protraction and elevation were also higher for the FSHD (no surgery) group participants. This was consistent with evaluation of overall scapulohumeral rhythm, with FSHD (no surgery) group participants demonstrating lower scapula rotation and tilts profiles. These results suggest that upper limb evaluations that focus on arm position alone are insufficient for explaining why people with FSHD lose arm function. People with FSHD demonstrated differences in the pattern, timing and level of normalized muscle activity. Increased normalized activity was seen most apparently in the trapezius, anterior deltoid and infraspinatus muscle groups. Further work is needed to evaluate the role of longitudinal measures, combined with additional imaging modalities to develop clinically informative methods.

## Disclaimers:

Funding: This work was funded by the Orthopaedic Institute Limited: Award RPG185.

Conflicts of interest: The authors, their immediate families, and any research foundation with which they are affiliated have not received any financial payments or other benefits from any commercial entity related to the subject of this article.
