# Supplementary material for: A study evaluating differences in 3D upper limb kinematics and surface electromyography measures in adults with and without facioscapulohumeral dystrophy
Source: JSES Rev Rep Tech. 2026 Jan 29;6(2):100670. doi: 10.1016/j.xrrt.2026.100670 (PMC12972986; doi:10.1016/j.xrrt.2026.100670)
Supplement: Supplementary Appendix S2 [file mmc2.pdf]

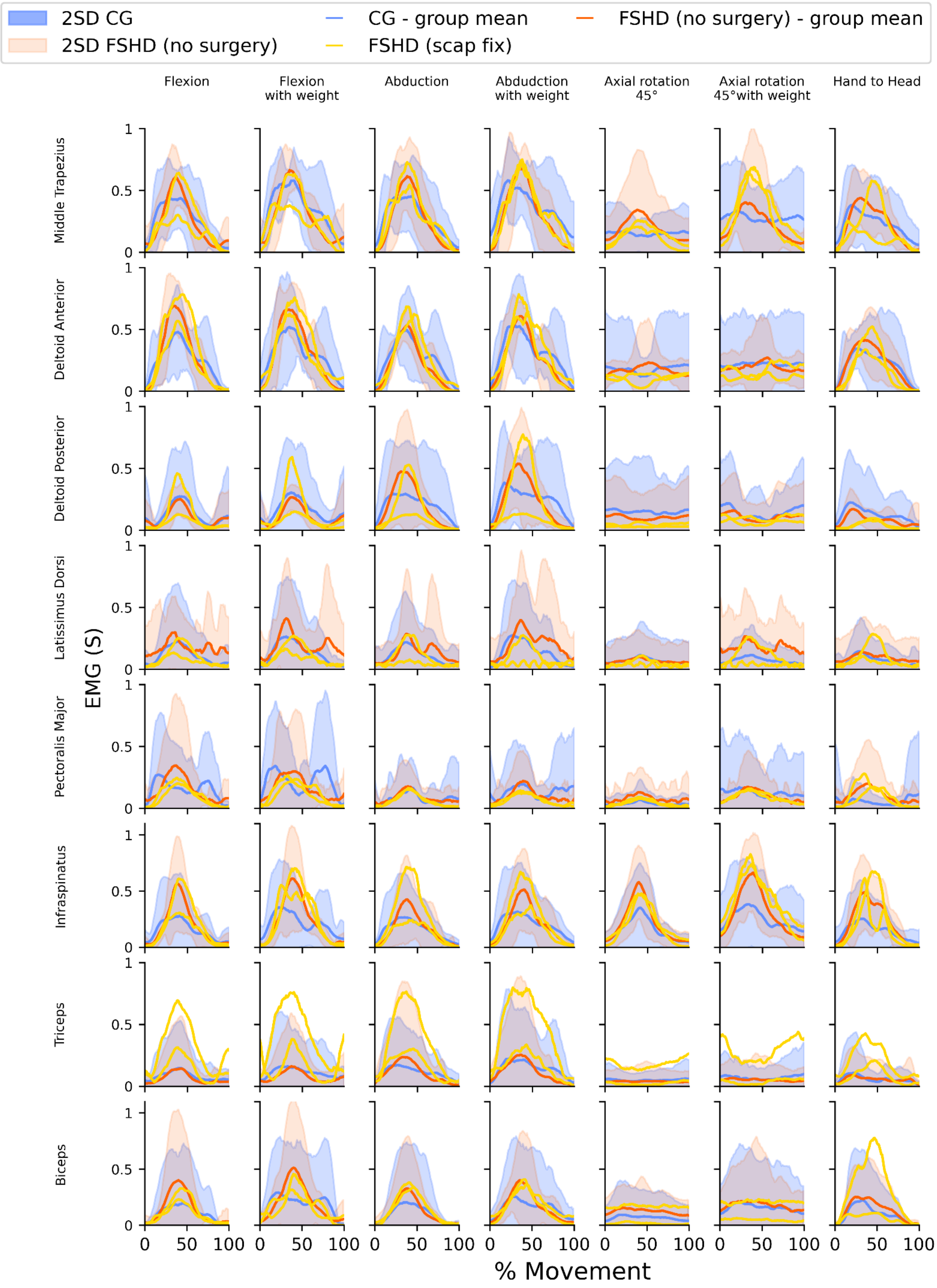

Surface EMG for all movements. Lines show mean normalised signals except for FSHD (scap fix) group, showing individual plots. Shaded areas indicate the 2SD. Column headings key: Axial rotation 45° – abduction at 45° with axial rotation; Axial rotation 45° with weight – abduction to 45° with axial rotation and weight; Hand to head – hand to back of head. FSHD (scap fix) – people with FSHD and previous scapular arthrodesis surgery; FSHD (No surgery) people with FSHD and no scapular arthrodesis; CG – age- and sex-matched control group; 2SD – 2 standard deviations.
